# Reminiscence groups for people with dementia and their family carers: pragmatic eight-centre randomised trial of joint reminiscence and maintenance versus usual treatment: a protocol

**DOI:** 10.1186/1745-6215-10-64

**Published:** 2009-07-30

**Authors:** Robert T Woods, Errollyn Bruce, Rhiannon T Edwards, Barry Hounsome, John Keady, Esme D Moniz-Cook, Martin Orrell, Ian T Russell

**Affiliations:** 1 Dementia Services Development Centre Wales, Institute of Medical and Social Care Research, Bangor University, Ardudwy Hall, Normal Site, Holyhead Road, Bangor, UK; 2 Bradford Dementia Group, University of Bradford, Unity Building, 25 Trinity Road, Bradford, UK; 3 Centre for Economics and Policy in Health, Institute of Medical and Social Care Research, Bangor University, Dean Street, Bangor, UK; 4 School of Nursing, Midwifery and Social Work, University of Manchester, University Place, Oxford Road, Manchester, UK; 5 Centre for Mental Health and Aging, Humber Mental Health Teaching NHS Trust, Coltman Street Day Hospital, 39-41 Coltman Street, Kingston-upon-Hull, UK; 6 Department of Mental Health Sciences, University College London, Wolfson Building, 48 Riding House Street, London, UK; 7 North Wales Organisation for Randomised Trials in Health and Social Care, Institute of Medical and Social Care Research, Bangor University, Ardudwy Hall, Normal Site, Holyhead Road, Bangor, UK

## Abstract

**Background:**

The growing number of people with dementia, and the increasing cost of care, provides a major incentive to develop and test methods of supporting them in the community for longer. Most attention has been given to pharmacological interventions, but there is increasing recognition that psychosocial interventions may be equally effective, even preferable where medication has negative side-effects. Reminiscence groups, run by professionals and volunteers, which use photographs, recordings and other objects to trigger personal memories are probably the most popular therapeutic approach to working with people with dementia, but there is little evidence for their effectiveness and cost-effectiveness. The recent inclusion of family carers in groups with people with dementia, notably in our own pilot studies, has generated informal evidence that this joint approach improves relationships between people with dementia and their carers, and benefits both.

**Design and methods:**

This multi-centre, pragmatic randomised controlled trial (RCT) to assess the effectiveness and cost-effectiveness of joint reminiscence groups for people with dementia and their family care-givers has two parallel arms – an intervention group and a control group who receive care as usual. The intervention consists of joint reminiscence groups held weekly for twelve consecutive weeks, followed by monthly maintenance sessions for a further seven months.

The primary outcome measures are the quality of life of people with dementia, as assessed by QoL-AD, and their care-givers' mental health as assessed by the GHQ-28. Secondary outcomes include: the autobiographical memories of people with dementia; the quality of the relationship between them and their care-givers; and the levels of depression and anxiety felt by them and their care-giver. Using a 5% significance level, comparison of 200 pairs attending joint reminiscence groups with 200 pairs receiving usual treatment will yield 80% power to detect a standardised difference of 0.38 in the QoL-AD rated by the person with dementia and 0.28 in the GHQ-28 or carer-rated QoL-AD. The trial will include a cost-effectiveness analysis from a public sector perspective.

**Discussion:**

Our Cochrane review (2005) on reminiscence therapy for people with dementia did not identify any rigorous trials or economic analyses in this field.

**Trial Registration:**

ISRCTN42430123

## Background

The development and evaluation of therapeutic interventions intended to benefit people with dementia and their family carers is the subject of much research interest at present. In view of the large and growing numbers of people with dementia, and the costs associated with meeting needs for care, there are clear advantages for health and social care services in supporting people with dementia in the community for longer but less intensively. However there is consensus that this should not be at the cost of additional burden on family carers [1].

Most attention has been given to pharmacological interventions, but there is increasing recognition that psychosocial interventions may have comparable value [2,3], and may be preferable in some contexts, e.g. where medication may be ineffective or have negative side-effects [3,4]. A number of systematic reviews of psychosocial interventions are now available [e.g. [1,5,6], as well as a number of Cochrane reviews of specific approaches [e.g. [7,8].

In the UK, Reminiscence Therapy appears to be the most well-known therapeutic approach to working with people with dementia. For example, over half of care homes in Wales claim to offer this approach to their residents [9]. Reminiscence work with people with dementia has an extensive history [10,11], engendering enjoyable activities that promote communication and well-being. One factor in its popularity is that it works with early memories, which are often intact for people with dementia, thus drawing on the person's preserved abilities, rather than emphasising the person's impairments. However, its popularity has not led to a corresponding body of evidence on its effects. The existing research literature has been brought together in our revised Cochrane review on reminiscence therapy for people with dementia [12]. Only four randomised controlled trials (RCTs) suitable for analysis were identified. Each examined different types of reminiscence work; all were small or of poor quality. The trials together identified significant improvements in cognition and mood 4–6 weeks after treatment, and stress in care-givers who participated with the person with dementia in a reminiscence group. However, the review concluded that 'in view of the limitations of the studies reviewed, there is an urgent need for more quality research in the field'. This dearth of evidence is reflected in the National Institute for Health and Clinical Excellence and Social Care Institute for Excellence (NICE-SCIE) Guideline on the management and treatment of dementia [3], which found insufficient evidence to recommend that reminiscence should be routinely offered to people with dementia, although its potential impact on mood of the person with dementia was highlighted.

To take research forward, there is a need to specify clearly the exact nature of the reminiscence work to be undertaken and its aims. Typically, a group approach has been implemented, with 'memory triggers' (photographs, recordings, artefacts etc.) used to promote personal and shared memories. A recent development has been to include family carers in reminiscence groups alongside their relatives with dementia. Descriptive evaluations suggest that this joint approach (described as 'Remembering Yesterday Caring Today' – RYCT [13]) may improve the relationship between carer and person with dementia, benefiting both [14]. As it is the breakdown of this care-giving relationship that increases the likelihood of the person with dementia being placed in an alternative care setting, such as a care home, this effect could have far-reaching implications for families, society and public spending. Our group have reported a very small pilot study evaluating this joint reminiscence approach (7 patient-carer pairs in the treatment group; 4 in the waiting-list control group), which showed some trends in improved quality of life for patients and reduced stress for care-givers [15]. In a larger trial platform, funded by the Medical Research Council (MRC), improvements in autobiographical memory and carer depression were associated with reminiscence groups containing 50 patient-carer pairs.

The justification for evaluating the joint reminiscence approach specifically comes from this promising pilot data and the great interest in this approach in the field of reminiscence work [10]. More generally, a recent meta-analysis [1] on interventions with family carers of people with dementia suggested that joint approaches may be more effective in improving carer outcomes than approaches targeted only at the carer. The previous tradition in dementia care of interventions for people with dementia and their carers separate from each other is being questioned. For example, in many areas of the UK, Alzheimer Café sessions have been established, with an agenda including education as well as social contact, attended by both people with dementia and their carers [16]. The emphasis has shifted from 'person-centred care' to 'relationship-centred care', with recognition of the central importance of the relationship between person with dementia and carer to the benefit of both [16]. Although a joint focus on people with dementia and their care-givers is not possible for all people with dementia, only 6% of them have no identifiable care-giver [17], and thus a higher chance of entering care homes.

The objective of this paper is to describe the study protocol of this pragmatic RCT among people with mild to moderate dementia and their family caregivers. The main research questions concern whether reminiscence groups with participants and carers followed by reminiscence-based maintenance are more (cost)-effective in ameliorating the quality of life of people with dementia and the stress on their carers than 'usual treatment'.

## Design and methods

### Design

**Figure 1:**
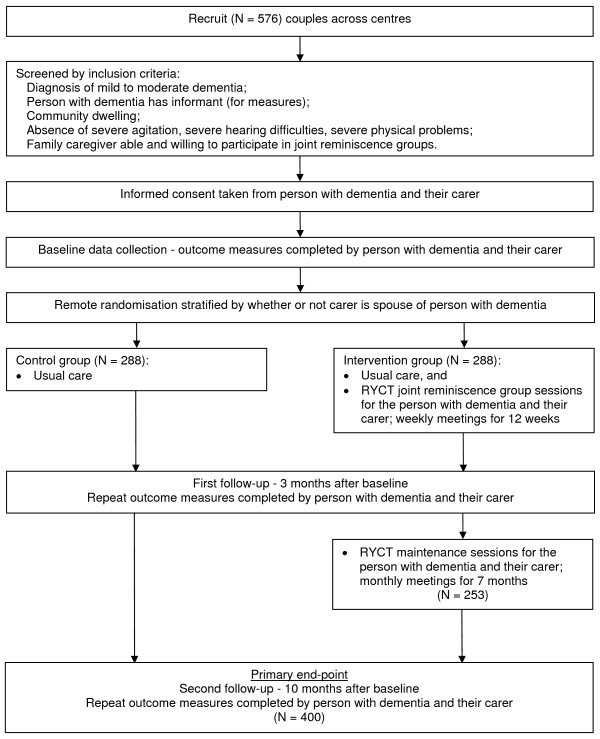
**Flow diagram for the trial**.

The design is a pragmatic eight-centre randomised trial (Figure 1). After selection, and baseline assessment, pairs of people with dementia and their family carer are randomised to one of two groups; the intervention group receives usual care plus joint reminiscence groups and reminiscence-based maintenance, the control group receives usual care only. Participants are free to seek additional assistance and support elsewhere at any time after baseline. Participants are allowed to enter the study only after giving signed informed consent in accordance with the provisions of the Mental Capacity Act 2005 [18]. General ethical approval was obtained through the Multi-centre Research Ethics Committee for Wales (ref no. 07/MRE09/58). The study is registered as a clinical trial with ISRCTN 42430123.

### Participants

Recruitment to this trial is taking place through mental health services for older people in each area (especially Memory Clinics, Community Mental Health Teams for Older People and associated professionals including psychiatrists, occupational therapists and Admiral Nurses), associated day services and through relevant local voluntary sector agencies such as the Alzheimer's Society and Age Concern. Recruitment will be in three waves, giving centres the opportunity to focus on different geographical areas in different waves. The Appendix sets out the inclusion and exclusion criteria for participants in the trial.

### Randomisation

Local researchers, who do not take part in follow-up assessments, contact the remote randomisation service of the North Wales Organisation for Randomised Trials in Health (NWORTH) when they have up to 24 pairs ready for randomisation. NWORTH is accredited as a Clinical Trials Unit by the UK Clinical Research Collaboration (UKCRC) and funded by the Clinical Research Collaboration Cymru, notably for HTA trials. The same researcher arranges for the 12 pairs randomised to the intervention group to attend sessions, and liaises with the group facilitator.

### Blinding

Though participants cannot be blinded to their allocated treatment, all follow-up data are gathered by blinded interviewers. However our experience in the trial platform, shared by similar projects, is that participants may occasionally and inadvertently inform researchers of the treatment they are receiving. We aim to reduce this effect by explicit reminders to participants before the assessment visit and by the use of self-report measures wherever feasible. We also ask all assessors to record their impression of the arm to which each participant belongs, and their confidence in that prediction. This will enable us to test whether inadvertent loss of blinding leads to bias, and to adjust for any bias detected.

### Intervention

The practice of using joint reminiscence groups attended by people with dementia and their carers [13] emphasises active, as well as passive, forms of reminiscence by both carers and the people with dementia. Pairs attend 12 two-hour sessions, where possible in a social not clinical setting. Each session focuses on a different theme, including childhood, schooldays, working life, marriage, and holidays and journeys. Couples are encouraged to contribute with materials brought from home. Each session blends work in large and small groups, and a range of activities including art, cooking, physical re-enactment of memories, singing and oral reminiscence. The inclusion of the person with dementia is paramount. In the joint reminiscence groups facilitators and volunteers guide carers to allow the person with dementia to respond and to value their contribution.

There is a limit of 12 pairs for the two trained facilitators in each group, supported by several trained volunteers. Our previous experience suggested that ideally these volunteers should cover a range of ages and come from the voluntary sector (e.g. Alzheimer's Society and Age Concern), health professional trainees and former carers with an understanding of working with older people. The training programme for facilitators and volunteers is set out in the RYCT manual, developed during the MRC trial platform [13]. Training engenders skills in listening, interpreting behaviours group dynamics, and enthusing carers and people with dementia. Two half-day training sessions take place before each group commences. After each session there is time for facilitators and volunteers to prepare session notes, complete attendance forms and collate evaluation forms. These evaluation forms are collected from carers and people with dementia at the end of the first session and at the end of the 12-week programme. The RYCT manual recommends a blend of activities for each session, based on core principles.

The presence of volunteers means that if, for any reason, carers are not able to attend all the group sessions, the person with dementia can still contribute to the group sessions. Subsequent maintenance sessions are held monthly and follow a similar pattern – re-visiting some topics and introducing new topics like a particular decade, for example the 1950s with the aid of relevant music and video clips.

### Usual care

The services and interventions available to people with dementia and family carers randomised to receive usual treatment will vary between and within centres and over time. In principle all the interventions offered to this group will also be available to those in the active treatment groups as we are evaluating the *additional *effects of reminiscence work. The only exception to this is when reminiscence groups occur at the same time as an alternative intervention. Our commitment to costing services and interventions received allows us to monitor whether control groups are receiving alternative interventions in this way. Though changes and developments in the availability of medications for Alzheimer's and other dementias should affect both groups equally, we intend to monitor this through the costing information we collect.

Participants in the usual treatment group may well engage in some form of reminiscence work during the 10 months of the study period. This is a popular approach in day-care centres, and reminiscence materials are widely available. However it is unlikely that structured reminiscence work will be offered in any of the centres, and even less likely that it will be offered jointly to carers. It is this systematic group-based approach, rather than a general exhortation to reminisce to improve communication, that is the focus of this evaluation.

### Ethical arrangements

#### Risks and anticipated benefits for trial participants

There appear to be no documented harmful side-effects from participating in reminiscence groups and no adverse reactions were apparent in the MRC trial platform. Some past memories can be unhappy, and even traumatic, but with a skilled and trained facilitator participants will share only those aspects they feel comfortable with, and if distressing memories were to surface, the person would be given additional support on a one-to-one basis.

Benefits are consistently reported by participants in the groups, including enjoyment, feelings of validation and self-worth. The desire of participants to continue meeting following the sessions provides an indication of the value placed on the benefits. Prospective participants will be fully informed of the potential risks and benefits of the project.

Nevertheless, a reporting procedure is in place to ensure that serious adverse events are reported to the Chief Investigator. Upon becoming aware of an adverse event involving a participant or carer, a member of the research team assesses whether it is "serious". A Serious Adverse Event (SAE) is defined in the trial as an untoward occurrence experienced by either a participant or carer which:

• results in death;

• is life threatening;

• requires hospitalisation or prolongation of existing hospitalisation;

• results in persistent or significant disability or incapacity;

• is otherwise considered medically significant by the investigator;

• falls within the scope of the Protection of Vulnerable Adults (POVA) protocol which is in place to ensure that suspected cases of abuse or neglect are followed-up in an appropriate manner.

A reporting form is submitted to the CI who assesses whether the SAE is:

• related to the conduct of the trial;

• unexpected.

SAEs that are judged to be related and unexpected are reported to MREC and the trial DMEC within 15 days.

#### Consent

Participants will be in the mild to moderate stages of dementia, and therefore would generally be expected to be competent to give informed consent for participation, provided that appropriate care is taken in explaining the research and sufficient time is allowed for them to reach a decision. In every case, the participant will have had at least 24 hours to consider the information provided. It is helpful for a family member or other supporter to be involved, and we would aim to ensure that this is done wherever possible. Informed consent will be sought separately from the family care-giver, in relation to their own participation. It will be made clear to both participants and family care-givers that no disadvantage will accrue if they choose not to participate.

In seeking consent, we will follow current guidance from the British Psychological Society on evaluation of capacity. In this context, consent has to be regarded as a continuing process rather than a one-off decision, and willingness to continue participating will be continually checked through discussion with participants during the assessments.

Where the participant's level of impairment increases, so that they are no longer able to provide informed consent, the provisions of the Mental Capacity Act will be followed, with the family care-giver as consultee. Where the person has themselves given informed consent initially, this provides a clear indication of the person's likely perspective on continuing at later time-points. The same procedure will apply where the person with dementia appears to lack capacity to consent initially, but meets the other criteria for the project. At any point where a participant with dementia becomes distressed by the assessments they will be discontinued.

### Outcome measures

Primary and secondary measures are completed at baseline, three months after baseline (first follow-up) and ten months after baseline (second follow-up and primary end-point). The interviews for the first follow-up are conducted after the completion of the weekly RYCT sessions, while the interviews for the second follow-up are scheduled after the final monthly maintenance session.

#### Primary outcome measures

a) Quality of life of the person with dementia, using the scale *Quality of Life in Alzheimer's Disease *(QoL-AD) [19], which covers 13 domains of quality of life. This is reliable and valid for people with mild and moderate degrees of dementia when they take part in structured interviews with trained interviewers [20,21].

b) Care-giver's mental health, assessed using the 28-item, self-completed *General Health Questionnaire *GHQ-28 [22] which has been widely used in care-giver research [23,24]; we shall use four-point Likert scales ranging from zero to three. The questionnaire includes indicators of anxiety, depression, insomnia, social dysfunction and somatic symptoms. We chose the GHQ-28 over the Relatives' Stress Scale because it is more general and more widely used.

#### Secondary outcome measures

a) Autobiographical memory, assessed using an extended version of the *Autobiographical Memory Interview *(AMI) [25]. The extended AMI assesses the person with dementia's recall of personal memories relating to both factual (semantic) information (for example, names of schools or teachers) and specific incidents. In the trial platform, we validated an additional section covering the period from middle-age to retirement, to cover the life-span of our participants.

b) Quality of relationship, assessed by both person with dementia and carer using the *Quality of the Carer-Patient Relationship *(QCPR) [26]. Originally developed in Belgium this scale comprises 14 items with 5-point Likert scales designed to assess the warmth of the relationship and the absence of conflict and criticism. In the trial platform the QCPR had good internal consistency for carers (α = 0.85) and for people with dementia (α = 0.80), and concurrent validity with other measures of relationship quality and carer stress.

c) Depression and anxiety, using *Cornell Scale for Depression in Dementia *(CSDD) [27] and *Rating Anxiety In Dementia *(RAID) [28] for person with dementia; and *Hospital Anxiety and Depression Scale *(HADS) [29] for carer. The CSDD is a 19-item scale, derived from interviews with the people with dementia and their carers in which the interviewer describes signs and symptoms to the carer. Where there is a discrepancy between carer's and clinician's ratings, the interviewer re-interviews the carer before making a final judgment. The RAID is an 18-item scale to rate anxiety in people with dementia based on structured interviews with them and their carers. The HADS is a well-validated 14-item, self-completed scale that measures both anxiety and depression, and is suitable for use with adults of all ages.

d) Stress specific to care-giving, using the *Relative's Stress Scale *[30], which asks the care-giver to complete 15 5-point Likert items.

e) Quality of life of person with dementia, rated by the care-giver using the proxy version of the *QoL-AD *[19], identical in structure and content to the version completed by the person with dementia.

f) General quality of life of both care-giver and person with dementia, using *EQ-5D *[31]. This is a standard measure of health-related utility, applicable to all health conditions. It provides a simple health profile and a single value for health status designed to calculate Quality Adjusted Life Years (QALYs). Care-givers will complete the measure from their own perspective and for the person with dementia, who will also complete it whenever possible.

g) Functional ability of person with dementia, using the *Bristol Activities of Daily Living Scale *[32], a 20-item scale completed by the carer.

h) Costs, using the validated *Client Services Receipt Inventory *(CSRI) [33]. Used extensively in studies of mental health and dementia (e.g. [34]), this gathers comprehensive data on accommodation, medication and services received. In this trial the data will reflect either the previous three months (at baseline and after treatment) or seven months (at follow-up).

#### Sample size

Our target sample size is 400 patients completing data collection for the trial after ten months, comprising 200 randomised to attend reminiscence groups and 200 receiving treatment as usual. In the trial platform intra-class correlation coefficients (ICCs) within randomised groups were often negative, but never significantly greater than zero, for both the carer-specific GHQ-28 and the carer-rated QoL-AD. However they were close to 0.1 for the QoL-AD rated by the person with dementia. Using a 5% significance level, therefore, comparison of the 200 pairs completing the intervention with the 200 pairs receiving treatment as usual will yield 80% power of detecting a standardised difference of 0.28 in carer-specific GHQ-28 or carer-rated QoL-AD. In contrast the patient-rated QoL-AD is likely to suffer a 'variance inflation factor' of approximately 1.74 [viz. 1 + 0.1 × (average completed group size of 8.4 minus 1)], thus yielding a power of 80% of detecting a standardised difference of 0.38. Our trial platform, which had a sample size of 57 in 3 centres, suggests that standardised differences of 0.28, even 0.38, are plausible. In our judgement they also fall within the range of clinically important effects. Furthermore, because our trial platform was exploratory, and therefore more heterogeneous than the proposed definitive trial, ICCs and standard deviations are likely to fall. To achieve a sample size of approximately 400, we allow for 12% attrition between recruitment and the post-treatment assessment (estimated from our trial platform) and a further 18% attrition over the following 7 months (estimated from a community study [35]). Hence we seek an initial sample size of 576, requiring 24 treatment groups initially comprising 12 pairs and another 288 pairs randomised to usual treatment.

### Analysis

#### Statistical Analysis of Effectiveness

We shall analyse by intention to treat and include all available data. However methods of imputing missing data such as last observation carried forward (LOCF) are of limited utility in dementia, where the norm is gradual decline until loss through death and illness. Hence our sample size calculations are based on the numbers we expect to be available at the study end-point, ten months after randomisation. We shall use multi-level modelling to address clustering within randomised groups. We shall use analysis of covariance to adjust for baseline differences in outcome variables. Analyses will treat ten months after randomisation as the primary end-point in evaluating whether the intervention has affected people with dementia or their care-givers. Secondary analyses will focus on effects immediately after the intensive group sessions.

#### Economic Analysis

The principal method will be cost-effectiveness analysis. We will use established methods for comparing costs and changes in scores of dementia specific quality of life instruments between the control and intervention groups [36]. There is a useful opportunity for secondary cost-utility analysis, more exploratory because estimating the utilities of people with dementia is neither easy nor very precise. To complete the picture, we plan to describe all costs and effects for people with dementia and their carers in a cost-consequence analysis.

#### Cost data

This analysis takes a public sector perspective spanning the NHS (dementia services, primary and secondary care) and local government. The interventions received will be fully costed from the perspective of local dementia services to generate a total programme cost and cost per participant-carer pair.

The measurement of health service use is the first step in estimating costs in economic evaluation. We shall estimate the costs of dementia care through the validated CSRI, completed by the family care-giver. There is a growing literature to support the reliability of patient recall as an alternative to GP records [e.g. [37] and our economic protocol is consistent with that used by previous trials in this field [33,34]. GP and other provider records are not an entirely accurate source of service use and hence costing information. These formal records, though mainly computer-based, are often incomplete or difficult to link between primary and secondary NHS care, and social services. So collecting data from GPs and other care providers for the whole sample would add little to the reliability of use and cost information in the planned evaluation. Nevertheless we propose to compare self-reported visits to primary and secondary care over the study period by a sample of 40 participants (20 experimental and 20 controls) with the corresponding GP notes to estimate systematic differences in reports. As the difference in costs and effects between groups is of primary interest, it is especially important to treat these groups identically when costing.

We shall use national unit costs [38,39] to convert service use to monetary costs, including:

• costs of running the joint reminiscence groups;

• costs of reminiscence-based maintenance groups following the initial intervention;

• direct costs of all health care used by participants in the intervention and control arms of the study, including contacts with GPs and practice nurses at home, in surgery or by phone, prescribing and outpatient and inpatient attendances); and

• indirect costs of lost productivity and care-givers attending group sessions;

but not intangible costs.

#### Effectiveness data

We shall evaluate effectiveness through the primary clinical outcomes – QoL-AD, the dementia-specific measure of quality of life and the carer-specific GHQ-28 – both at the primary end-point of 10 months.

#### Incremental Cost-effectiveness Analysis

Incremental cost-effectiveness ratios (ICERs) will summarise changes in the costs and effects of improving quality of life of people with dementia and stress in their care-givers through joint reminiscence group therapy followed by reminiscence-based maintenance, compared with usual care. We shall use bootstrapping to characterise the uncertainty in the cost-effectiveness analysis by estimating the probability distribution of ICERs and generating cost-effectiveness acceptability curves (CEACs) to quantify and display that uncertainty. Though CEACs are widely used in economic evaluation of health care technologies [40], they are no less useful in the evaluation of psycho-social interventions.

### Sensitivity analysis

We shall use sensitivity analyses to assess whether assumptions underpinning economic analyses affect the results of those analyses. For example, we shall assess how collecting data for only 10 months and assuming no differential effects beyond that point, affects ICERs and CEACs relative to assumptions like differences at 10 months decline to zero over next 10 months.

#### Secondary Cost-Utility Analysis

We shall conduct an exploratory cost-utility analysis using EQ-5D to calculate QALYs for carers and people with dementia, if necessary by getting carers to complete the EQ-5D 'by proxy' for people with dementia [41,42]. The addition of EQ-5D to interview schedules for both care-givers and people with dementia will enable us to estimate and potentially combine the health utility gains to both people with dementia and their carers. This responds to the recommendations from NICE to include utility measures in trials of new drugs and interventions to facilitate cost per QALY calculation and consider the health effects of an intervention regardless of who receives them: "For the reference case, the perspective on outcomes should be all direct health effects whether for patients or, where relevant, other individuals (principally carers)" [43]. Dixon et al have recently highlighted the potential influence on cost per QALY ratios of adding the health utility gains of carers to those of people with conditions like dementia [42]. Given the findings of the trial platform, we predict that both the costs and effects of reminiscence therapy may be modest, with the result that the cost per QALY ratio may have a large standard error. Added to our concern about using a generic measure of quality of life for people with dementia, this leads us to label the cost-utility analysis as secondary to the cost effectiveness analysis.

#### Cost-Consequence Analysis

Cost-consequence analysis estimates the incremental costs and consequences of alternative programmes without aggregation. In particular our analysis will compare secondary outcomes of experimental and control participants at baseline, 3 months, and 10 months, thus elaborating on the summative cost effectiveness and cost utility analyses by addressing a wider range of costs and consequences arising from reminiscence therapy. This will help commissioners and policy-makers responsible for funding and coordinating services.

## Discussion

This paper summarises the study protocol of an innovative and pragmatic RCT that evaluates the effectiveness and cost-effectiveness of joint reminiscence groups and maintenance sessions for people with dementia and their carers. Our Cochrane review [12] generated this, the first rigorous trial of joint reminiscence groups and one of the first integrated cost-effectiveness studies of a psycho-social intervention for dementia. With its emphasis on working with the person with dementia and family carer together, our therapeutic approach reflects current emphasis on 'relationship-centred care', and our trial will accurately estimate the outcomes for both parties. The NICE-SCIE guidelines on the management of dementia offered few evidence-based recommendations on psychosocial approaches because there were few good studies. The results of this trial will contribute to future updates of these guidelines, so that the approach becomes widely used in health and social care, if successful in this trial.

## Competing interests

The authors declare that they have no competing interests.

## Authors' contributions

Contributions: RTW, MO & EB developed the original concept for the trial, and RTW drafted the original protocol; ITR developed the design and methodology; RTE developed the health economic component; EB co-authored the treatment manual; BH & RTE adapted the trial proposal as a protocol paper; all authors reviewed and commented on drafts of the protocol and paper.

## Appendix

### Inclusion and exclusion criteria

#### Inclusion criteria

All participants are people with dementia who:

○ meet the DSM-IV criteria for dementia of any type, including Alzheimer's, vascular, Lewy Body type and mixed;

○ are in the mild to moderate stage of dementia (Clinical Dementia Rating);

○ can communicate and understand communication, shown by a score of 1 or 0 on the relevant items of the Clifton Assessment Procedures for the Elderly – Behaviour Rating Scale;

○ can engage in group activity;

○ live in the community at the time of the baseline assessment and have a relative or other care-giver who maintains regular contact, can act as informant, and is willing and able to participate in the intervention with the person with dementia.

#### Exclusion criteria

Participants do not have any characteristic which could affect participation, e.g.:

○ major physical illness;

○ sensory impairment;

○ disability; or

○ high level of agitation.
